# Dr. Malden's Fatal Cases of Bronchocele

**Published:** 1826-07

**Authors:** 


### Bronchocele.*

As bronchocele, comparatively speaking, but seldom destroys life, a brief account of some fatal cases of this disease, as related by Dr. Malden, may not prove uninteresting.

Dr. M. observes that great danger results from any tumour on the

* Dr. Malden, of Worcester, Med. Repos. April, 1826.

sides of the trachea, on account of its compression by the sterno-cleido and other muscles of the neck, which from their frequent action are extremely liable to obstruct the respiration. Bronchocele may often be discovered by the difficulty of breathing, asthmatic paroxysms, &c. when it is not marked externally. When there may be visible only a general fulness, or lateral bulging of the neck, pressure on both sides will bring on wheezing or even a sense of suffocation, which will detect at once the deep-seated tumour. With regard to the question of how the impediment to respiration is caused, it is obvious that it must be principally from direct pressure, but in a great measure also from irritation.

*Case* 1. Mary Silsey, ætat. 18, entered the Worcester Infirmary, June 19th, 1819. Her respiration had been difficult for more than a year, but was of late much worse; she had constant wheezing, slight cough, with, at times, a little mucous expectoration; pulse small and quick. The tumour extended, on the left side of the neck, from the angle of the jaw down to the sternum, and had a firm and knotty feel; the carotid of that side was stretched behind it, and communicated strong pulsation to it—on the right side, the swelling was more even and not so large. Leeches at first gave relief; then blistering was tried; and finally a seton on the left side; but without effect—headaches now attacked her; and in December she died, after a severe paroxysm of dyspnœa and head-ache.

*Dissection.* Thyroid gland much enlarged; the left lobe extended upwards, and under the muscles of the neck, which were on the stretch; it was very firm and adhered to a chain of white steatomatous tumours, of the size of hazel-nuts which passed down under the sternum, and terminated below the bifurcation of the trachea, half an inch of which was compressed, and its cavity nearly obliterated by the enlarged thyroid gland. One of the steatomatous tumours also caused considerable pressure on the trachea, at its bifurcation—this tube and the bronchiæ were filled with a colourless frothy fluid; mucous membrane sound; lungs healthy.

*Cranium.* The veins of the pia mater, sinuses, and jugulars full of blood; half an ounce of fluid in the ventricles; pons varolii much softened, and of a dirty yellow colour.

*Remarks.* Although in this case, steatoma was added to the bronchocele, yet the latter certainly made most pressure on the respiratory tube. The gorging of the venous system of the head depended principally on the obstruction to the flow of blood in the internal jugular, and partly on the difficulty of breathing.

*Case* 2. Dec. 7th, 1821. Eliza J. ætat. 32, in the last month of her third pregnancy, was affected with bronchocele of several years duration, which had increased during both her former pregnancies, and very fast of late. It extended from the sternum to the jaw; the sterno-cleido muscles were stretched over it; the carotids were forced back and felt pulsating on a line with the transverse processes of the cervical vertebræ; the superior thyroid arteries large, and beating with a peculiar thrill; dyspnœa extreme; the chin convulsively raised at each inspiration, which was stridulous; and the face anxious and suffused with blood. She died of gradual suffocation, Dec. 10th.

*Dissection.* The thyroid gland was much enlarged, passing deep on each side of the trachea and bound down by the muscles above-mentioned. When cut into, the tumour was not very firm; it was of a pink colour, and contained a thick gelatinous matter in innumerable small cells. *An inch and a half of the trachea was so compressed, as to be quite flattened on the sides, and to present in front a sharp edge*: its mucous membrane was slightly inflamed, and filled with a muco-purulent fluid.

*Remarks.* Bronchocele frequently attacks for the first time during pregnancy, and then its growth is rapid. If it has arisen before pregnancy, then, upon that taking place, its growth is often much accelerated, and, according to Dr. M., " though it greatly subsides after delivery, it never, without medical aid, resumes the size it had before the pregnancy of the patient, so that each succeeding pregnancy adds a permanent increase to the enlargement."

In young females, a bronchocele will suddenly grow very fast, and subside upon the establishment of the catamenia.

*Case* 3. Mr. —— ætat. 50, short and rather stout, had been subject for many years to fits of dry cough, increased by exertion, and resembling asthma. Eight or nine years ago he observed a fulness in his throat, particularly on the left side, which increased as well as the dyspnœa and cough. August 10th, 1825, Dr. M. first saw him; his breathing was laborious, with loud wheezing; his position was that of an asthmatic, the shoulders being fixed, and the chest forcibly expanded: he had suffered thus for many weeks, and in these attacks the countenance became quite livid.

On the left side of the throat was found a tumour, firm and elastic above, softer below, reaching from the angle of the jaw to the clavicle and sternum, and forcing the larynx three quarters of an inch, or more, to the opposite side. On the right of the trachea was another tumour, smaller and moveable. Behind these enlargements the carotids were easily traced, giving them a strong pulsation. Towards the last, he was delirious at times, and on August 29th, he perished from suffocation.

*Dissection.* The left lobe of the thyroid gland was greatly enlarged, and consisted of several large cysts, containing a gelatinous substance of various degrees of consistence. This lobe passed into the chest as low as the arch of the aorta; and its lowest cyst, which contained an ounce and a half of a glairy fluid, was lodged between the innominata and left carotid. The internal jugular veins, par vagum, and carotid arteries were much pushed backwards; and three quarters of an inch of the trachea, just below the cricoid cartilage, were rendered nearly impervious. The muscles above the tumours were stretched, and the cellular membrane, under the platysma, excessively dense and strong. The mucous membrane of the trachea and bronchi was somewhat inflamed: the lungs, heart, and abdominal viscera sound.

*Case* 4. Mr. ——, of Kempsey, ætat. 17, had been subject to attacks of dyspnœa for four years, generally brought on by exertion. There was unusual fulness of the throat; and pressure made, at the same time, on both sides of the neck, brought on wheezing, dyspnœa, and distress. After considerable exertion, he was seized with a very severe paroxysm, since which the heart had pulsated with great force and frequency. Blood was taken from the arm; leeches repeatedly applied to the throat; the sulphate of magnesia given in small doses every four hours, with ten drops of tinct. digitalis. The symptoms of excitement soon subsided, and ten drops of tinct. iodinæ were given three times a day. Under this treatment the tumour soon disappeared, and with it the dyspnœa, &c. nor have they since returned.

*Remarks.* This case exemplifies the good effects of iodine in the early stage of bronchocele, as also the insidious nature of that complaint.

